# *Eucalyptus globulus*: From Botanical Identity to Cross-Disciplinary Preclinical Relevance in Dentistry, Medicine, and Nanotechnology

**DOI:** 10.3390/plants15142121

**Published:** 2026-07-09

**Authors:** Oana Raluca Radbea, Ştefania Dinu, Daniel Raul Chioibas, Andra Ardelean, Cosmin Sinescu, Bogdan Hoinoiu, Ramona Amina Popovici, Sergio Liga, Diana Florina Nica

**Affiliations:** 1 Doctoral School, “Victor Babeș” University of Medicine and Pharmacy, Eftimie Murgu Square No. 2, 300041 Timisoara, Romania; raluradbea@gmail.com; 2 Department of Pediatric Dentistry, Faculty of Dental Medicine, “Victor Babeș” University of Medicine and Pharmacy, Eftimie Murgu Square No. 2, 300041 Timisoara, Romania; dinu.stefania@umft.ro; 3 Department of Surgery I, Faculty of Medicine, “Victor Babeș” University of Medicine and Pharmacy, Eftimie Murgu Square No. 2, 300041 Timisoara, Romania; chioibas.raul@umft.ro; 4 University Clinic of Dentofacial Aesthetics, Faculty of Dental Medicine, “Victor Babeș” University of Medicine and Pharmacy, Eftimie Murgu Square No. 2, 300041 Timisoara, Romania; 5 Department of Prosthetic Technology and Dental Materials, “Victor Babes” University of Medicine and Pharmacy, 9 Revolutiei 1989 Ave., 300070 Timisoara, Romania; sinescu.cosmin@umft.ro; 6 University Clinic of Oral Rehabilitation and Dental Emergencies, Faculty of Dentistry, “Victor Babeș” University of Medicine and Pharmacy, Eftimie Murgu Square No. 2, 300041 Timisoara, Romania; 7 Department I—Management and Communication, Faculty of Dental Medicine, “Victor Babeș” University of Medicine and Pharmacy, Eftimie Murgu Square No. 2, 300041 Timisoara, Romania; ramona.popovici@umft.ro; 8 University Clinic of Toxicology, Drug Industry, Management and Legislation, Faculty of Pharmacy, “Victor Babes” University of Medicine and Pharmacy, Eftimie Murgu Square No. 2, 300041 Timisoara, Romania; sergio.liga96@gmail.com; 9 Department of Applied Chemistry and Engineering of Organic and Natural Compounds, Faculty of Chemical Engineering, Biotechnologies and Environmental Protection, Politehnica University Timisoara, Vasile Pârvan No. 6, 300223 Timisoara, Romania; 10 Department of Anesthesiology and Oral Surgery, Faculty of Dental Medicine, “Victor Babeş” University of Medicine and Pharmacy, Eftimie Murgu Square No. 2, 300041 Timisoara, Romania; nica.diana@umft.ro

**Keywords:** *Eucalyptus globulus*, essential oil, preclinical relevance, dentistry, medicine, nanotechnology

## Abstract

*Eucalyptus globulus* is a widely cultivated medicinal species of recognized phytochemical richness and broad preclinical relevance across multiple biological and formulation-oriented application domains. This review aimed to integrate the available evidence on the botanical identity, phytochemical profile, preclinical biomedical relevance, and translational considerations within a single structured framework. A bibliometric-assisted narrative review approach was applied, based on targeted literature retrieval from scientific platforms (e.g., PubMed, ScienceDirect, MDPI, Google Scholar), complemented by keyword co-occurrence analysis and qualitative narrative synthesis. The reviewed literature indicates that *E. globulus* is characterized by a 1,8-cineole-rich essential oil together with non-volatile phenolic, flavonoid, and related secondary metabolites that support a wide range of reported biological activities. The strongest preclinical evidence concerns antimicrobial and antibiofilm effects in oral-health-related models, antioxidant and anti-inflammatory activity, respiratory relevance, wound-healing support, selected cytotoxicity-related effects, and emerging nano-enabled delivery systems. Overall, *E. globulus* represents a promising multifunctional species with substantial preclinical potential; however, direct clinical evidence remains limited, and future progress will depend on better phytochemical standardization, mechanistic clarification, and well-designed translational studies.

## 1. Introduction

Medicinal plants continue to occupy an important place in both traditional and modern healthcare systems, being recognized as rich sources of structurally diverse bioactive compounds with broad therapeutic potential [1,2]. Medicinal and aromatic plants are also described as a major reservoir of secondary metabolites (e.g., terpenoids, essential oils, phenolic compounds, alkaloids, related phytochemicals), which underpin a wide spectrum of biological activities and therapeutic applications [2,3]. Recent review literature further emphasizes that these botanical resources are relevant not only to health care, but also to the pharmaceutical, food, cosmetic, perfumery, and other industrial sectors, reflecting their expanding functional and economic significance [3]. At the same time, several reviews note that a substantial proportion of the global population still relies on herbal or traditional plant-based medicine for primary health care, particularly in settings where conventional medical infrastructure remains limited or less accessible [2,3]. Contemporary interest in medicinal plants has been further intensified by the growing burden of antimicrobial resistance, as plant-derived compounds are increasingly investigated as potential antimicrobial agents and as alternative sources of bioactive molecules for drug discovery [1,4]. However, the literature also highlights persistent challenges related to poor bioavailability, limited stability, standardization, quality control, sustainability, and regulatory oversight, which have directed current research toward advanced delivery systems and more integrated translational approaches [2,3,4].

Within the large area of research on medicinal and aromatic plants, *Eucalyptus globulus* (hereafter abbreviated as *E. globulus*) has attracted particular attention as a widely cultivated species of recognized medicinal and industrial value. It is described as one of the most widely utilized medicinal plants and as the principal global source of eucalyptus leaf oil, owing to its widespread cultivation, essential oil yield, and the predominance of 1,8-cineole in its phytochemical profile [5,6]. The available scientific literature attributes to *E. globulus* a broad range of reported biological and practical applications, including antibacterial, antifungal, anti-inflammatory, antioxidant, respiratory, dental, insecticidal, phytopathogen-control, food-preservative, and phytoremediation-related uses [6,7,8]. Experimental studies further indicate that *E. globulus* essential oil may exert marked activity against oral pathogens and biofilms, while more recent work has examined its antioxidant, anti-inflammatory, antibacterial, antibiofilm, and anticancer-related properties through phytochemical and bioactivity profiling [7,9]. At the same time, the reviewed articles note that a comprehensive and systematic evaluation of eucalyptus essential oils remains necessary, and that convincing clinical trial evidence for eucalyptus essential oils and cineole is still limited [5,9]. Beyond its medicinal relevance, *E. globulus* also has substantial economic and applied importance as one of the most widely cultivated eucalyptus species. Its broad dissemination has been supported by its adaptability to different environmental conditions, rapid growth, woody biomass production, and high essential-oil value. In addition to being recognized as a major source of eucalyptus leaf oil, *E. globulus* has attracted sustained industrial interest because of its relevance for woody biomass and plantation-based production systems, while eucalyptus-derived products also remain important in pharmaceutical, hygiene, cosmetic, and related application sectors [3,6].

In this context, a focused review on *E. globulus* is justified to integrate the available information on its botanical identity, phytochemical composition, reported biological properties, and emerging therapeutic relevance within a single structured framework.

## 2. Scope of the Review and Methodology

The aim of this review was to examine *Eucalyptus globulus* as a medicinally relevant species by integrating evidence on its botanical identity, phytochemical profile, preclinical relevance, and translational considerations within a single structured framework. Specifically, the review covers: (i) the botanical aspects of *E. globulus*; (ii) its phytochemical profile, with emphasis on essential oil composition and major secondary metabolites; (iii) preclinical relevance across dentistry, medicine, and nanotechnology-oriented applications; and (iv) current clinical and translational limitations.

This manuscript was designed as a bibliometric-assisted narrative review and not as a PRISMA-based systematic review. The review process combined structured literature retrieval, bibliometric mapping, and qualitative narrative synthesis. The workflow involved five main stages: (i) identification of relevant peer-reviewed publications; (ii) organization and cleaning of bibliometric records; (iii) descriptive and relational processing of the dataset; (iv) bibliometric visualization of thematic structures; and (v) integration of the retrieved evidence into a narrative synthesis aligned with the main sections of the review.

The literature search was conducted in PubMed [10], ScienceDirect [11], MDPI [12], and Google Scholar [13], covering publications from January 2020 to March 2026. These sources were selected for their broad multidisciplinary coverage of biomedical, phytochemical, pharmaceutical, and plant science literature. To capture complementary and foundational evidence, the reference lists of relevant reviews and key primary studies were also screened for earlier publications considered scientifically important for contextualizing the field. The search strategy was based on combinations of terms related to the species name, phytochemistry, and the main thematic domains addressed in the review. The principal search terms included “*Eucalyptus globulus*”, “eucalyptus essential oil”, “1,8-cineole”, “phytochemical profile”, “antimicrobial”, “dentistry”, “oral pathogens”, “respiratory”, “anti-inflammatory”, “wound healing”, “anticancer”, and “nanotechnology”. These terms were applied in different combinations depending on the database and the thematic focus of each search.

Studies were considered eligible for discussion when they met the following criteria: (i) they were peer-reviewed publications; (ii) *Eucalyptus globulus* was the primary focus of the study, or the article provided clearly relevant supportive evidence in a comparative Eucalyptus-based context; (iii) the publication addressed at least one of the themes covered in this review, including botanical aspects, phytochemistry, dentistry-oriented relevance, medical or pharmaceutical preclinical evidence, nanotechnology-enabled systems, or translational considerations; and (iv) the study provided sufficient methodological and interpretive detail for qualitative discussion. Publications were excluded when they were not sufficiently relevant to the scope of the review, lacked adequate methodological detail, focused on unrelated non-biomedical or non-phytochemical topics, or represented duplicate records across databases. After retrieval, records were screened for topical relevance, and duplicates were removed where necessary. Studies retained for the narrative synthesis were selected based on their direct relevance to the review scope, their methodological clarity, and their contribution to the thematic sections of the manuscript. Because this article was developed as a bibliometric-assisted narrative review rather than as a formal systematic review, the screening process was intended to improve transparency of evidence selection without following a PRISMA-based workflow.

**Figure 1:**
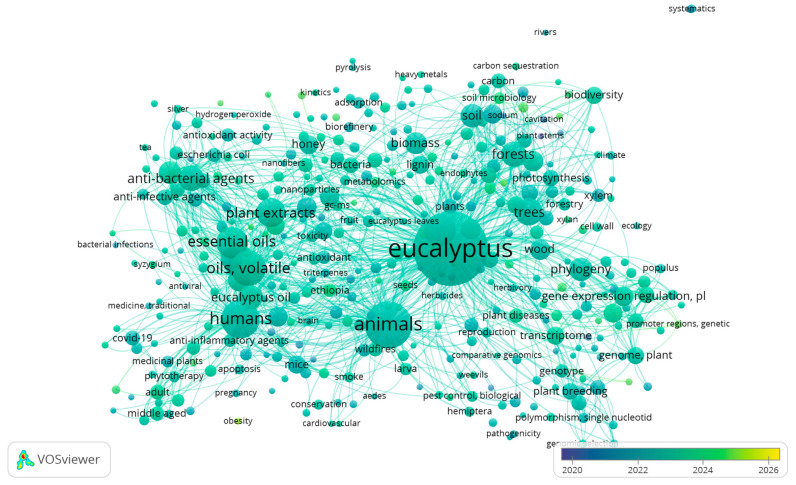
Co-keyword occurrence network (overlay visualization) constructed in VOSviewer from all keywords of the retrieved scientific publications (January 2020–March 2026).

A keyword co-occurrence analysis was performed to identify the main thematic structures and research trends in publications related to *E. globulus*. For the bibliometric component, keyword co-occurrence analysis was performed using VOSviewer v1.6.20 [14], based on a PubMed-derived dataset comprising 1708 publications retrieved within the predefined time (January 2020–March 2026). This corpus was used specifically for thematic mapping and visualization of the conceptual structure of the field. In parallel, the narrative synthesis was developed from a thematically selected subset of studies retrieved from PubMed, ScienceDirect, MDPI, and Google Scholar, together with earlier relevant references identified through citation screening. Because the analysis includes all extracted keywords, the network also contains broader indexing terms; therefore, Figure 1 is intended primarily for thematic mapping rather than causal inference.

The co-occurrence map indicates that the *E. globulus* literature is organized around several interconnected thematic domains, with eucalyptus as the central and most prominent node. One major thematic core is centered on essential oils, volatile oils, eucalyptus oil, plant extracts, and related terms, highlighting the strong emphasis of the field on phytochemical composition and bioactivity-oriented research. A second domain appears to reflect biomedical and experimental relevance, as indicated by the presence of broad indexing and application-related terms, including humans, animals, and health-oriented concepts. Taken together, these patterns suggest that the literature on *E. globulus* is structured primarily around the relationship between phytochemical characterization and experimentally explored biological activity, rather than around clinically consolidated application areas.

**Figure 2:**
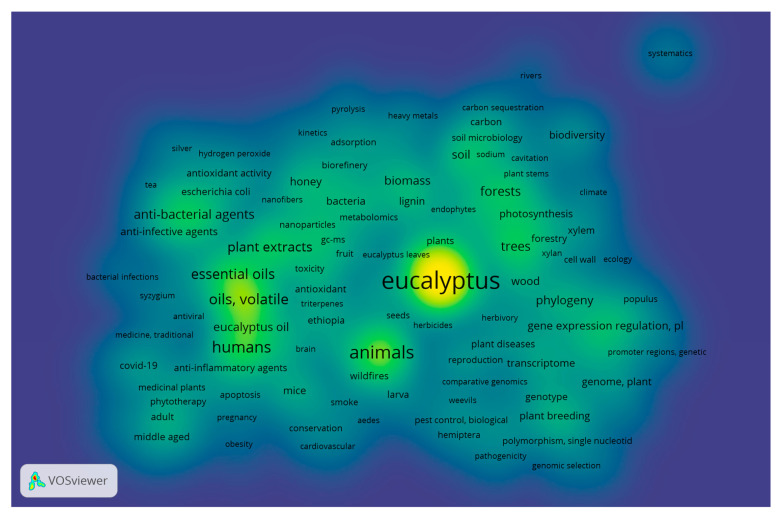
Density map visualizing keywords from scientific publications (January 2020–March 2026).

The density visualization further supports the observation that thematic evolution in this field has been relatively gradual rather than sharply discontinuous (Figure 2). The most prominent nodes remain associated with established phytochemical and bioactivity-related topics, while fewer highly frequent terms appear to be strongly associated with the most recent publication years. This pattern may indicate that recent research has largely expanded existing lines of investigation rather than generating clearly dominant new thematic clusters. Because the network was generated from all extracted terms, including broad indexing keywords, these findings should be interpreted primarily as indicators of thematic structure and evolution rather than as direct measures of research impact or causal priority.

Under these conditions, a bibliometric-assisted narrative approach was considered more suitable than a formal systematic review framework for integrating conceptually diverse evidence within a single structured discussion. This approach offers the advantage of broader thematic synthesis and field-level mapping, but it also has limitations, including a lower degree of procedural standardization and a greater potential for selection bias than a PRISMA-based systematic review. In the present manuscript, the bibliometric analysis and the narrative synthesis were conducted as complementary but non-identical components: (i) the bibliometric mapping was used primarily to identify thematic structures and support field scoping, whereas (ii) the narrative synthesis was developed independently through thematic selection and qualitative interpretation of studies relevant to the review scope.

## 3. *Eucalyptus globulus* Labill.: Botanical Aspects and Phytochemical Profile

### 3.1. Botanical Aspects

*E. globulus* Labill. is an accepted species of the family Myrtaceae and the order Myrtales. It is commonly referred to as blue gum or *Tasmanian bluegum* [15,16]. It is native to southern Victoria and eastern Tasmania, Australia, but beyond its native range, it has been extensively cultivated worldwide owing to its adaptability to environmental conditions, rapid growth, woody biomass production, and essential oil value [6,17]. *E. globulus* is an evergreen broadleaf tree with a straight trunk and is commonly described as a large species with smooth, grayish-blue, exfoliating bark and slightly ridged branchlets [6,18]. The species shows marked foliar dimorphism. Juvenile leaves are opposite, sessile, and amplexicaul, with an ovate oblong to cordate shape, whereas adult leaves are alternate, petiolate, lanceolate, thick, and elongated [18]. Mature leaves have been reported to reach up to 25 cm in length and 1.7–3 cm in width, while other descriptions characterize them as shiny dark green, narrowly lanceolate, and borne on yellowish petioles [6,18]. The reproductive morphology is also diagnostically relevant, with axillary inflorescences arranged in simple umbels that may be one- or seven-flowered, supported by flattened or terete peduncles. The combination of exfoliating bluish bark, heteromorphic foliage, elongated aromatic adult leaves, and characteristic umbellate inflorescences contributes to the morphological identification of *E. globulus* within the genus *Eucalyptus* [18,19]. From a pharmacobotanical perspective, accurate species authentication remains important in the case of eucalyptus-derived raw materials, particularly because medicinal and commercial products may involve more than one taxon within the broader eucalyptus group. For this reason, correct identification of *E. globulus* based on macroscopic and botanical features remains a relevant prerequisite for subsequent phytochemical interpretation and quality assessment.

### 3.2. Phytochemical Profile

**Figure 3:**
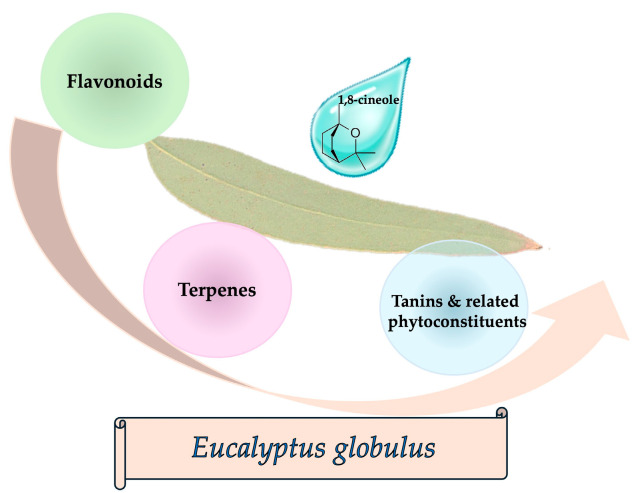
Phytochemical profile of *E. globulus*.

The phytochemical profile of *E. globulus* Labill. is primarily characterized by its essential oil-rich leaves, with 1,8-cineole generally reported as the dominant constituent [1,2,5,6]. In addition to this major volatile compound, the species contains a range of other monoterpenes, sesquiterpenes, phenolic compounds, flavonoids, tannins, and related secondary metabolites [2,5,6]. Overall, the available evidence indicates that the biological relevance of *E. globulus* is associated with a chemically diverse profile, including both volatile and non-volatile constituents (Figure 3).

#### 3.2.1. Major Volatile Constituents of the Eucalyptus Essential Oil

The volatile fraction of *E. globulus* is dominated by monoterpenes, particularly oxygenated monoterpenes, with 1,8-cineole (eucalyptol) consistently reported as the principal constituent of the leaf essential oil [5,6]. Representative volatile constituents reported for *E. globulus* leaf essential oil in studies included in the narrative synthesis are summarized in Table 1, highlighting the predominance of cineole-rich profiles in most scientific reports.

The studies summarized in Table 1 indicate that the essential-oil composition of *E. globulus* may vary substantially across reports. This variability likely reflects differences in plant material, geographical origin, developmental stage, extraction procedure, and analytical approach. Although 1,8-cineole predominates in most studies, some reports show marked shifts toward other monoterpenes or sesquiterpenoid-rich profiles, indicating that phytochemical comparisons across studies should be interpreted with caution. The available literature indicates that oil recovery may vary according to multiple factors (e.g., geographical origin, environmental conditions, physiological stage, extraction-related variables). Therefore, the phytochemical profile of *E. globulus* should be interpreted not only in terms of the relative abundance of individual volatile constituents, but also in relation to the factors that may influence the quantity of oil obtained under different conditions.

Although the present discussion focuses primarily on leaf essential oil, it should also be noted that volatile profiles may differ among plant organs, including flowers, fruits, bark, and other aerial parts. Such organ-dependent variation may influence the ecological role, biological activity, and potential industrial relevance of eucalyptus-derived volatile fractions. Nevertheless, because the available literature on *E. globulus* remains much more strongly centered on leaves and leaf oil than on other organs, the present review gives priority to the phytochemical evidence derived from leaf material.

#### 3.2.2. Non-Volatile Constituents

Beyond its volatile essential oil fraction, *E. globulus* has also been reported to contain a broader range of non-volatile phytoconstituents, particularly in leaf extracts. This broader phytochemical spectrum indicates that the biological relevance of *E. globulus* cannot be interpreted solely based on its essential oil composition. Qualitative screening reported by Lad et al. confirmed the presence of triterpenoids, flavonoids, phenols, and steroids, while quantitative analysis reported total phenolic and total flavonoid contents of 65.12 ± 0.01 mg GAE/g and 57.75 ± 0.01 mg QE/g, respectively [9]. Evidence from extract-based studies also provides greater detail on individual non-volatile compounds. In a study of the ethanolic leaf extract, HPLC analysis identified kaempferol, quercetin, and myricetin among the detected phytochemicals, with kaempferol showing the highest relative abundance, followed by myricetin and quercetin [24]. The currently available evidence on non-volatile constituents remains more limited and less standardized than that describing the volatile fraction, but it consistently supports the presence of polyphenolic and related secondary metabolites as relevant components of the phytochemical profile of *E. globulus*.

## 4. Preclinical Research and Cross-Disciplinary Relevance of *Eucalyptus globulus*

The phytochemical profile of *E. globulus*, particularly its cineole-rich essential oil together with its broader range of secondary metabolites, underpins its relevance across multiple application domains, including dentistry, medicine, and nanotechnology. This cross-disciplinary interest is closely related to the reported antibacterial, antibiofilm, anti-inflammatory, antioxidant, and formulation-oriented potential of the species, which has supported its investigation in oral care, respiratory and topical applications, and bioactive delivery systems. At the same time, the available evidence is not uniformly distributed across these domains, with some applications being supported primarily by experimental and formulation-based studies rather than by robust clinical validation. Unless otherwise stated, priority is given in the following sections to studies directly investigating *E. globulus*, whereas evidence derived from other *Eucalyptus* species or mixed eucalyptus-based formulations is included only as supportive context.

### 4.1. Dentistry-Oriented Applications and Preclinical Evidence

#### 4.1.1. Antimicrobial and Antibiofilm Relevance in Oral Health

Within the present review, antibacterial activity is supported most directly by studies targeting cariogenic biofilms, plaque-related *Streptococcus mutans* models, and periodontal pathogens. Overall, the available evidence suggests that eucalyptus-derived preparations may act at different levels of the biofilm process, including planktonic growth inhibition, reduction in biofilm biomass, interference with adhesion-related models, and incorporation into oral delivery systems such as films and mouthwash-type formulations.

In a study conducted by Goldbeck et al., the essential oils of *E. globulus* and *Eucalyptus urograndis* were evaluated against planktonic cells and biofilms of *Streptococcus mutans*, the microorganism considered a key etiological agent in dental caries. The authors reported inhibition zones ranging from 14.7 ± 1.2 mm to 35.3 ± 0.34 mm, a reduction of 1 log cycle in cell viability within less than 5 min of contact, and inhibition of *S. mutans* biofilm formation. Among the two oils, *E. globulus* showed the best performance, requiring only 15 min to induce microbial death, whereas *E. urograndis* required 50 min. Importantly, the antibiofilm effect of both oils was reported to be greater than that of 0.1% sodium fluoride, and antimicrobial starch films containing *E. globulus* oil also reduced *S. mutans* counts after 4 h of contact. The authors attributed the stronger performance of *E. globulus* mainly to its higher 1,8-cineole content [7]. Similarly, in a study conducted by Landeo-Villanueva et al., commercially available essential oils of *Mentha spicata* and *E. globulus* were tested against *S. mutans* ATCC 25175 in an in vitro model designed to emulate dental plaque conditions. The *E. globulus* oil was characterized by 1,8-cineole (65.83%) and α-pinene (18.15%) as the main constituents. Using agar-well diffusion and colorimetric microdilution, the authors found an inhibition halo of 27.0 ± 0.82 mm and an MIC of 1.9168 mg/mL. In the bovine enamel/basal mucin model with daily sucrose exposure cycles, application of the oils at 0.5% significantly reduced biofilm biomass and the number of recoverable organisms after 72 h, confirming activity not only against planktonic *S. mutans* but also under biofilm-forming conditions that more closely resembled oral plaque development [25].

In a study conducted by Kumar et al., a blended oil-pulling preparation containing virgin coconut oil, eucalyptus oil, peppermint oil, thyme oil, and clove oil was tested against the periodontal pathogen *Porphyromonas gingivalis* and compared with chlorhexidine. The authors reported that *P. gingivalis* was sensitive to the blended essential-oil preparation at MIC values of 100 μg/mL and 50 μg/mL, whereas chlorhexidine inhibited growth at 0.4 μg/mL. Although eucalyptus was only one component of the blend and the study did not isolate its individual contribution, the paper remains relevant to this subsection because it extends the antimicrobial discussion from cariogenic plaque models toward periodontal pathogens and supports the potential role of eucalyptus-containing mixtures in plaque-associated oral infections [26].

In a study conducted by Batista et al., a eucalyptus essential oil-based nanoemulsion prepared from *E. globulus* and *Eucalyptus citriodora* was developed for mouthwash applications and evaluated against *Streptococcus mutans*. The nanoemulsion showed an average particle size of around 100 nm, a polydispersity index close to 0.3, zeta potential values between −19 and −30 mV, and cell viability above 50% in rabbit surface mucous cells. According to the authors, the nanoemulsion was effective in controlling *S. mutans*, and mouthwash formulations functionalized with the nanoemulsion displayed both bacteriostatic and bactericidal properties. This study is particularly relevant because it connects antimicrobial efficacy with a realistic oral-care vehicle and suggests that eucalyptus-based nanoformulations may support preventive dentistry applications beyond free essential oils [27]. In a study conducted by de Souza et al., chitosan films containing distilled pyroligneous extracts of Eucalyptus grandis were evaluated for oral applications against *Candida albicans*, *Streptococcus mutans*, and *Lactobacillus acidophilus*. Although the study did not investigate *E. globulus*, it remains relevant as supportive eucalyptus-based evidence within oral biofilm control. The authors reported antimicrobial activity by direct contact test, differences in demineralization-related outcomes across test groups, and no statistically significant differences in cytotoxicity relative to the control in NIH/3T3 cells. They proposed that these eucalyptus-containing chitosan films, particularly in the form of an experimental fluoride varnish, may inhibit oral microbial colonization and help prevent biofilm formation related to dental caries [28]. In a study conducted by Balkrishna et al., an oil-pulling formulation named Dant-Kanti-Gandush, containing a blend of essential oils from clove, peppermint, *E. globulus*, prickly ash, and basil in coconut and sesame oil, was evaluated against oral pathogens and inter-kingdom biofilms on orthodontic fixtures. The authors reported MIC50 values ranging from 0.10% to 0.45% (*v*/*v*) against *Streptococcus pyogenes*, *Streptococcus mutans*, *Proteus mirabilis*, and *Candida albicans*. At concentrations ≥ 1.0 × MIC50, the formulation impaired cariogenic traits of *S. mutans* by reducing biofilm formation, acid production, and survival under acidic stress. In addition, it inhibited biofilm formation and hyphal transition in *C. albicans*, and scanning electron microscopy showed reduced microbial density, disrupted bacterial aggregation, and fragmented fungal hyphae on orthodontic fixtures. Because this was a blended formulation, the results cannot be attributed to *E. globulus* alone; however, the study is highly relevant to oral-health antibiofilm research because it expands the discussion from single-species cariogenic models to mixed bacterial–fungal biofilms formed on dental devices [29].

Additional supportive evidence broadens the interpretation of the antimicrobial and antibiofilm relevance of eucalyptus-derived preparations, although these studies are not strictly based on oral plaque models. In a study conducted by Iseppi et al., *E. globulus* essential oil showed activity against antibiotic-resistant bacterial biofilms, particularly during biofilm formation, and displayed synergistic interactions when combined with tea tree oil or antibiotics [30]. In addition, Polito et al. reported antibiofilm activity for several *Eucalyptus* species, supporting the broader anti-biofilm potential of the genus [31], while Mezzasalma et al. demonstrated that *E. globulus* leaf extract, as well as ursolic and asiatic acids, exerted antibacterial and partial biofilm-inhibitory effects against biofilm-producing bacterial isolates [32].

Taken together, the strongest direct evidence in this subsection concerns *E. globulus* itself in *Streptococcus mutans*-related and oral-care-oriented models, whereas the remaining studies provide broader supportive context for eucalyptus-based antibiofilm relevance. A recurring pattern across studies is that *E. globulus*-based preparations appear more informative when evaluated under biofilm-forming or application-relevant conditions than in simple planktonic assays alone, particularly when incorporated into delivery systems (e.g., films, mouthwash-type nanoemulsions, complex oil-based preparations). At the same time, direct comparison across studies remains difficult because the tested materials differ substantially in composition, concentration, formulation type, and degree of species specificity. Thus, although the overall direction of evidence is supportive, the current literature remains methodologically heterogeneous and still limited in terms of standardized oral-health models directly focused on *E. globulus*.

#### 4.1.2. Oral Pathogens and Plaque-Related Models

Beyond general antimicrobial and antibiofilm effects, additional studies have examined *E. globulus* in oral pathogen-oriented and plaque-related models, particularly in relation to periodontal relevance and clinically observable gingival outcomes. In a study conducted by Müller-Heupt et al., the antimicrobial efficacy of *E. globulus* leaf extract against *Porphyromonas gingivalis* ATCC 33277 was assessed using broth microdilution according to CLSI standards. The work was performed in the context of periodontitis, where *P. gingivalis* is regarded as a major periodontal pathogen and where plant-derived local antimicrobials are being explored because of the limitations and side effects of currently used antiseptics such as chlorhexidine. They reported measurable antimicrobial activity of *E. globulus* extract against *P. gingivalis*, with minimal inhibitory concentrations falling within the range of 64 to 1024 mg/L. Although the strongest antimicrobial effect in the comparative design was observed for *Rheum palmatum* rather than for *E. globulus*, the results still support the relevance of *E. globulus* as a plant-derived antimicrobial candidate in periodontal pathogen-oriented models [33]. In a study conducted by Osman et al., the efficacy of a eucalyptus oil-based dentifrice was evaluated in a randomized crossover clinical design involving participants with gingivitis. Although this study falls outside the strictly preclinical domain, it remains relevant to plaque-related oral models because it examined plaque index and gingival bleeding as clinically observable outcomes. Both tested herbal toothpastes significantly reduced plaque scores, while the eucalyptus oil-based toothpaste showed a greater reduction in gingival bleeding compared with miswak. These findings provide preliminary clinical support for the plaque- and gingivitis-related relevance of eucalyptus-based oral care formulations [34].

These studies indicate that *E. globulus*-derived preparations have also been investigated in plaque-related and periodontal-oriented models, extending their dentistry-related relevance beyond antibiofilm activity alone and toward gingival and oral-pathogen-focused outcomes; however, direct evidence remains limited and includes only a small amount of preliminary human data.

#### 4.1.3. Endodontic Pathogens and Local Disinfection Approaches

In addition to plaque- and periodontal-related models, eucalyptus-derived products have also been investigated in dentistry-oriented settings relevant to endodontic infection control and local disinfection. In these contexts, the available scientific literature has focused mainly on *Enterococcus faecalis*, a persistent pathogen frequently associated with root canal treatment failure, as well as on the disinfection of endodontic instruments and intracanal environments.

In a study conducted by Balhaddad and AlSheikh, eucalyptus oil was tested against *Enterococcus faecalis* using a 24 h biofilm model. The authors reported that diluted eucalyptus oil significantly reduced both total bacterial growth and biofilm formation compared with the untreated control. Mean total absorbance decreased from 0.63 in the control to 0.02 in the eucalyptus oil group, while biofilm formation was reduced by approximately 30-fold. These findings suggest that eucalyptus oil may have adjunctive value in preventing the growth of endodontically relevant pathogens [35]. In a study conducted by Agarwal et al., eucalyptus was evaluated as one of several herbal root canal irrigants against persistent root canal pathogens, particularly *Enterococcus faecalis*. The study compared different natural products, especially eucalyptus, with conventional irrigants including sodium hypochlorite and chlorhexidine. According to the authors, the herbal products showed antibacterial effectiveness against *E. faecalis*, while the highest antibacterial effectiveness overall was observed for sodium hypochlorite, chlorhexidine, and eucalyptus extract. Within this comparative design, eucalyptus therefore emerged as one of the more active herbal alternatives tested for endodontic irrigation [36].

Supportive evidence from other *Eucalyptus*-based studies further broadens the interpretation of endodontic relevance, although such reports should be distinguished from direct *E. globulus* evidence. In a study conducted by Raoof et al., methanolic extracts of several medicinal plants were tested against *Enterococcus faecalis*, *Porphyromonas gingivalis*, and *Fusobacterium nucleatum*. Across all concentrations and time intervals studied, eucalyptus extract showed the highest antimicrobial activity against *E. faecalis*. The authors also found that all tested extracts inhibited the growth of *P. gingivalis* and *F. nucleatum*, and that no significant differences were observed between the anti-enterococcal effects of eucalyptus extract and non-ready-to-use calcium hydroxide. Although this study investigated *Eucalyptus galbie* rather than *E. globulus*, it remains relevant as supportive eucalyptus-based evidence for antimicrobial potential in endodontic pathogen models [37].

In a study conducted by Opi et al., eucalyptus oil was evaluated as a disinfecting agent for endodontic K-files contaminated with microorganisms of endodontic origin, including *Enterococcus faecalis*, *Staphylococcus aureus*, *Streptococcus β-hemolyticus*, *Escherichia coli*, *Pseudomonas aeruginosa*, *Peptostreptococcus* spp., and *Bacteroides fragilis*. The study compared tea tree, ajwain, eucalyptus, and neem oils using turbidity testing, serial dilution colony counts, and microscopic examination. Although eucalyptus oil was not the best-performing agent in this comparative design, it still demonstrated antimicrobial activity and was discussed as a potentially useful adjunct for the disinfection of endodontic instruments [38].

Taken together, these studies indicate that eucalyptus-derived products have been investigated not only in oral biofilm and plaque-related models, but also in endodontic contexts involving persistent pathogens and local disinfection strategies. The available evidence supports their potential relevance for adjunctive root canal infection control, although results vary depending on the formulation tested, comparator agents, and experimental model used.

### 4.2. Medical and Pharmaceutical Preclinical Evidence

#### 4.2.1. Antioxidant and Anti-Inflammatory Activities

Antioxidant and anti-inflammatory effects represent some of the most relevant pharmacological properties attributed to *E. globulus*, particularly in preclinical models characterized by oxidative imbalance, inflammatory activation, and tissue dysfunction. In a study conducted by Moreira et al., both the essential oil and the hydrodistillation residual water extract of *E. globulus* showed anti-inflammatory effects in LPS- and Aβ-stimulated microglia, reducing the expression of pro-inflammatory mediators such as Nos2, IL-1β, IL-6, TNF-α, and PTGS2/COX-2. In the same study, the hydrodistillation residual water extract also reduced intracellular oxidative stress and attenuated mitochondrial dysfunction in neuronal models, while intranasal administration of both extracts in APP/PS1 mice reduced cortical and hippocampal Aβ levels and improved behavioral outcomes, supporting a combined antioxidant and anti-inflammatory effect in an Alzheimer’s disease context [39]. In a study conducted by Naranjo-Viteri et al., *E. globulus* essential oil, mainly composed of 1,8-cineole, was evaluated in a 6-hydroxydopamine rat model of Parkinson’s disease. The treatment improved spatial memory, motor performance, and grip strength, while also increasing TH and DAT protein content in the caudate-putamen. From the inflammatory perspective, the authors reported reduced hippocampal TNF-α expression in eucalyptus-treated parkinsonian rats, indicating that the neuroprotective effect of the essential oil may be associated, at least in part, with modulation of inflammatory signaling [40].

Evidence from systemic toxicity models further supports the antioxidant relevance of *E. globulus*. In a study conducted by Mousa et al., a methanolic leaf extract of *E. globulus* protected male rats against diclofenac sodium-induced hepatic, renal, and testicular toxicity. This protection was associated with restoration of reduced glutathione levels in testicular tissue, together with improvement of biochemical and histopathological parameters, suggesting that the extract counteracted diclofenac-induced oxidative injury [41]. Supportive in vivo evidence was also reported by Mohebodini et al., who found that dietary eucalyptus essential oil increased serum superoxide dismutase and reduced malondialdehyde in broiler chickens [42], and by Mohebodini et al., who showed that eucalyptus essential oil increased SOD and GPx activities, elevated nitric oxide levels, and reduced MDA concentrations in cold-stressed broilers with ascites-related oxidative disturbances. At the phytochemical level, these biological effects are consistent with the antioxidant potential of *E. globulus* phenolic-rich extracts [43]. In a study conducted by Lima et al., optimized extraction of *E. globulus* leaves yielded phenolic-rich extracts containing gallotannin, quercetin, and isorhamnetin derivatives, and the authors described these extracts as having strong antioxidant properties [44]. Similarly, Santos et al. reported that water ultrasound-assisted extracts obtained from *E. globulus* leaves and branches displayed antioxidant properties and could be applied in functionalized textile systems, further supporting the bioactive potential of eucalyptus-derived phenolic fractions [45].

Taken together, the available evidence indicates that the antioxidant and anti-inflammatory relevance of *E. globulus* is supported across multiple preclinical contexts, but not with equal strength. The most mechanistically informative studies are those conducted in neuroinflammation- and oxidative stress-related models, where *E. globulus* extracts or essential oil were associated with modulation of inflammatory mediators, reduction in oxidative imbalance, and improvement of functional or behavioral outcomes. By contrast, evidence from toxicity models, poultry studies, and extract-optimization studies is supportive but more indirect from a translational biomedical perspective. Overall, the literature suggests that the biological activity of *E. globulus* in this domain is plausible and recurrent, although comparisons across studies remain limited by substantial differences in model systems, phytochemical preparations, and outcome measures.

#### 4.2.2. Respiratory Relevance

Respiratory and airway-related use represents one of the most established therapeutic contexts associated with eucalyptus-derived preparations. As summarized by Mieres-Castro et al., eucalyptus essential oils, particularly those rich in 1,8-cineole, have long been used in respiratory conditions such as the common cold, nasal congestion, sinusitis, bronchitis, asthma, influenza, acute respiratory distress syndrome, and chronic obstructive pulmonary disease. The same review emphasizes anti-inflammatory, mucolytic, spasmolytic, and expectorant effects as key contributors to this respiratory relevance and identifies *E. globulus* as one of the principal medicinal sources of eucalyptus essential oil [46]. Mechanistic antiviral support for airway-related relevance was provided by Fernandez et al., who showed that eucalyptus essential oil and eucalyptol significantly reduced infection by a SARS-CoV-2 Spike pseudotyped lentivirus in hACE2-transfected 293T cells, with IC50 values of 0.00895% and 0.0042% (*v*/*v*), respectively. The authors also observed inhibition of vesicular stomatitis virus pseudovirus, suggesting that the antiviral effect was at least partly independent of Spike-mediated entry. Although this model does not fully reproduce respiratory tissue infection, it supports the concept that eucalyptus-derived volatiles may interfere with viral infection relevant to the respiratory tract [47]. Additional support was reported by Chen et al., who investigated eucalyptus essential oil against porcine reproductive and respiratory syndrome virus in vitro. While no clear protective effect was observed in pre-treatment or post-treatment settings, the oil showed marked virucidal activity during co-treatment, strongly reducing viral infectivity and, after sufficient co-incubation time, abolishing it. These findings suggest that eucalyptus essential oil may act directly on free viral particles rather than primarily through intracellular antiviral mechanisms [48].

The respiratory relevance of *E. globulus* is supported primarily by long-standing pharmacological positioning and by mechanistic preclinical evidence, particularly in antiviral and inflammation-related airway models. However, the available scientific studies differ substantially in terms of tested preparations, target systems, and biological endpoints, and the current evidence remains much stronger at the mechanistic and preclinical level than at the level of direct translational validation.

#### 4.2.3. Wound-Healing Effect

Wound-healing and tissue-related effects of *E. globulus* have been investigated mainly in infection-associated wound models and in formulation-based studies designed to improve local tissue repair. In a study conducted by Jiang et al., non-volatile fractions obtained from *E. globulus* fruit residues were evaluated against methicillin-resistant Staphylococcus aureus and vancomycin-resistant enterococci in vitro and in vivo. The 60% and 95% ethanol extracts inhibited both MRSA and VRE with MIC values of 8 μg/mL, disrupted biofilms, and altered bacterial surface morphology. In a mouse MRSA-infected wound model, the authors further reported that the active fraction promoted wound recovery, with better healing than the untreated model and a concentration-dependent reduction in bacterial load. Histologically, treated wounds showed improved tissue organization relative to the model group, supporting the view that *E. globulus* fruit-derived bioactive components may contribute to infected wound repair through combined antibacterial and tissue-restorative effects [49].

Additional formulation-based evidence supports the broader tissue-repair relevance of eucalyptus-derived preparations, although these studies should be interpreted as supportive rather than species-specific evidence for *E. globulus*. In a study conducted by Elbhnsawi et al., nano-chitosan/eucalyptus oil/cellulose acetate electrospun nanofibers were developed and evaluated for antimicrobial and wound-healing potential. Because the oil source in this study was *Eucalyptus camaldulensis*, the findings should be regarded as supportive eucalyptus-based evidence rather than direct evidence for *E. globulus*. The authors reported enhanced antimicrobial activity after encapsulation, particularly against Staphylococcus aureus, together with acceptable in vitro cell viability and improved in vivo healing outcomes associated with increased expression of TGF-β1 and type I and type III collagen [50]. A related infection-oriented study by El-Sakhawy et al. evaluated eucalyptus oil and a mixed-oil preparation in *Candida albicans*-infected wounds in rats. Although the work did not specifically involve *E. globulus*, it provides additional supportive evidence for eucalyptus-containing topical systems in infection-delayed wound repair. The mixed-oil formulation showed higher antifungal activity than eucalyptus oil alone and, when applied as a 10% cream, produced greater wound contraction, a shorter epithelialization period, lower fungal burden, and better histopathological recovery than the untreated infected group [51].

Similarly, Tahmasebi and Yazdanian evaluated a collagen–propolis–eucalyptus hydrogel in a rat wound model. Because the eucalyptus source was not specified as *E. globulus*, this study should also be considered supportive rather than direct evidence. The hydrogel showed no significant cytotoxicity, exhibited antibacterial activity against *Staphylococcus aureus* and *Escherichia coli*, and was associated with greater wound contraction and improved histopathological repair relative to the comparison groups [52].

These studies indicate that eucalyptus-derived preparations may contribute to wound healing not only by reducing microbial burden, but also by supporting tissue restoration under conditions in which infection compromises normal repair. However, the direct evidence specific to *E. globulus* remains more limited than the broader eucalyptus-based supportive literature, and comparison across studies is constrained by marked differences in formulation design, infection models, and biological endpoints.

#### 4.2.4. Cytotoxic Effects and Anticancer Activity

The anticancer-related potential of *E. globulus* has been investigated both at the level of the essential oil and through structurally defined non-volatile constituents, particularly formyl-phloroglucinol-derived metabolites. The available evidence suggests that *E. globulus* may exert moderate and, in some cases, selective cytotoxic effects against cancer cells, although the strength of evidence remains predominantly in vitro and varies substantially according to the phytochemical fraction studied.

Khazraei et al. evaluated *E. globulus* essential oil in SW48 colon cancer cells, HepG2 liver cancer cells, HEK293t cells, and human fibroblasts. The results showed that SW48 viability decreased in a concentration-dependent manner, with significant effects at 0.05%, 0.1%, 0.5%, and 5%, while 0.01% did not reduce SW48 viability. The reported IC50 values were 0.2% for SW48 and 5% for fibroblasts, whereas HepG2 and HEK293t cells showed no toxicity except at the higher dose range, with an IC50 of 0.2% reported for both. The same study also showed that 0.1% EGEO induced 13.8% cell death in SW48 cells and 13.0% in fibroblasts after 48 h, compared with 28.6% and 20.8%, respectively, for 100 μg/mL 5-FU. On this basis, the authors considered 0.05% after 48 h to provide the most favorable antiproliferative effect on SW48 cells without detectable fibroblast toxicity, while AO/PI staining further supported apoptosis-related morphological changes in treated SW48 cells [53]. A similar pattern was observed by Lad et al., who examined *E. globulus* essential oil in B16F10 melanoma cells and L929 fibroblasts using 200–1000 μL/mL for 24 h. The oil showed a concentration-dependent but moderate effect on melanoma cells, with inhibition increasing from 10.14% at 200 μL/mL to 41.44% at 1000 μL/mL. By contrast, inhibition in L929 fibroblasts remained lower, ranging from 4.73% to 16.89% over the same concentration interval. The 5-fluorouracil control was markedly more potent, with inhibition values from 38.51% to 85.81%. These findings suggest that the essential oil has only modest anticancer activity, although with comparatively limited toxicity toward normal fibroblasts [9].

In a study conducted by Wang et al., two new phloroglucinols, eucalyptals D and E, together with the related compound euglobal-In-3, were isolated from *E. globulus* fruits. All three compounds exhibited significant in vitro cytotoxicity against Huh-7, Jurkat, BGC-823, and KE-97 cancer cell lines, indicating that fruit-derived phloroglucinol-sesquiterpene adducts may represent more active antitumor constituents than the essential oil itself [54]. This pattern was reinforced by Liu et al., who studied formyl-phloroglucinol meroterpenoids from *E. globulus* leaves. Their petroleum ether extract showed cytotoxicity against MDA-MB-231, 786-O, and HepG2 cells, with IC50 values of 21.67 ± 2.26, 12.18 ± 0.70, and 17.15 ± 1.31 μg/mL, respectively. Among the isolated compounds, eucalypglobulusal L (2), eucalypglobulusal H (8), and eucalrobusone C (10) showed moderate cytotoxicity, with IC50 values ranging from 8.18 to 23.13 μM. Mechanistically, these compounds elevated ROS, malondialdehyde, and lipid ROS levels in MDA-MB-231 cells at 6.25–25 μM, while compounds 8 and 10 also reduced intracellular GSH. In addition, all three downregulated Nrf2, and compound 2 specifically inhibited GPX4 expression, supporting a redox-dependent mechanism linked to lipid peroxidation and impaired antioxidant defense [55].

Overall, the current literature suggests that the anticancer-related profile of *E. globulus* is not uniform across its phytochemical fractions. The essential oil itself appears to exert only moderate and partly selective antiproliferative effects, whereas the more structurally defined non-volatile phloroglucinol-derived constituents provide stronger and mechanistically more informative cytotoxic evidence. A recurring pattern across the reviewed studies is that the most convincing anticancer-related activity is associated with isolated meroterpenoid or phloroglucinol-type compounds rather than with the crude oil. At the same time, this body of evidence remains almost entirely in vitro and is based on highly heterogeneous cell models and preparations, which limits direct comparison and precludes firm translational interpretation.

### 4.3. Nanotechnology-Enabled Delivery and Functionalization Applications

Nanotechnology-enabled approaches involving *E. globulus* have been explored mainly to improve the stability, delivery, and functional performance of eucalyptus-derived bioactives, especially essential oil-based systems (Table 2). The available studies show that research in this area has focused primarily on nanoencapsulation, micellar and oil-in-water nanoemulsions, biopolymer-assisted formulations, and, to a lesser extent, bioactive metal-based nanoparticles synthesized using *E. globulus* extracts or essential oil. Across these systems, the main rationale has been to overcome the intrinsic limitations of essential oils and plant-derived compounds (e.g., volatility, poor aqueous dispersibility, instability, limited persistence), while enhancing biological activity in antifungal, antimicrobial, antibiofilm, repellent, analgesic, and drug-delivery contexts.

These studies indicate that nanotechnology-based strategies may extend the applicability of *E. globulus* beyond the use of crude extracts or free essential oil by enabling more stable, better-dispersed, and functionally enhanced formulations. Nevertheless, the current evidence remains heterogeneous and largely preclinical, and most studies are still focused on formulation development and proof-of-concept biological assays rather than on clinically validated applications.

## 5. Clinical Evidence and Translational Considerations

Despite the broad preclinical literature on *E. globulus*, direct clinical evidence remains limited and is still insufficient to support strong species-specific therapeutic conclusions. Much of the available human literature concerns eucalyptus oil, 1,8-cineole/eucalyptol, or proprietary eucalyptus-derived preparations rather than chemically standardized *E. globulus* formulations, which complicates direct comparison across studies and weakens species-level clinical attribution. In addition, most of the evidence summarized in the present review derives from in vitro studies, animal experiments, or formulation-based investigations, whereas robust human studies remain scarce. This imbalance is particularly relevant from a translational perspective, because promising biological effects observed in preclinical systems cannot be directly extrapolated to clinical efficacy without better standardization of the tested preparations and clearer disease-oriented validation.

Overall, the main translational limitations include variability in phytochemical composition, inadequate standardization of extracts and essential oils, differences among crude extracts, isolated compounds, and nanoformulations, and the scarcity of randomized controlled trials with clinically meaningful endpoints. Accordingly, although *E. globulus* shows clear preclinical promise across oral, respiratory, anti-inflammatory, wound-related, cytotoxicity-related, and formulation-oriented contexts, its therapeutic relevance in humans should still be interpreted with caution. Better-defined and chemically standardized clinical studies are needed before firm species-specific therapeutic recommendations can be made.

## 6. Conclusions

*E. globulus* emerges from the available literature as a botanically well-defined and phytochemically rich species whose biological relevance extends across dentistry, medicine, and nanotechnology-oriented research. Its therapeutic interest is supported by a chemically diverse profile dominated by 1,8-cineole-rich essential oil together with non-volatile phenolic, flavonoid, and related secondary metabolites, which together underpin a broad range of reported bioactivities. Across the preclinical literature reviewed here, *E. globulus* has shown particularly consistent relevance in antimicrobial and antibiofilm models, antioxidant and anti-inflammatory settings, respiratory and wound-related applications, and selected cytotoxicity-oriented studies, although the strength of evidence varies substantially depending on the extract type, formulation, and experimental model used. In parallel, nanotechnology-enabled systems (e.g., nanoemulsions, micellar formulations, polymer-assisted encapsulation platforms, bioactive nanoparticles) suggest that eucalyptus-derived compounds may benefit from improved stability, delivery, and functional performance when incorporated into advanced formulations. The currently available evidence supports *E. globulus* as a promising multifunctional species with substantial preclinical potential; however, the translational gap remains considerable, as most reported benefits are still supported predominantly by in vitro studies, animal models, or formulation-based investigations rather than by robust clinical validation. Future progress will depend on greater phytochemical standardization, clearer mechanistic characterization, translationally relevant formulation strategies, and well-designed clinical studies capable of validating its therapeutic relevance in humans.

## Figures and Tables

**Table 1:** Major volatile constituents reported for *E. globulus* in studies employing different extraction and analytical approaches.

Extraction Analysis	Major Phytochemical Constituents Reported	References
Hydrodistillation	1,8-cineole reported as the major constituent; leaf essential oil commonly described as containing 20–54 components	[6]
Steam distillation; GC-MS	1,8-cineole (71.05%), α-pinene (8.30%)	[7]
Steam distillation; GC-MS	Eucalyptol (51.62%), α-pinene (23.62%), p-cymene (10.00%), β-myrcene (8.74%), terpinen-4-ol (2.74%), γ-terpinene (2.59%)	[20]
GC-MS	1,8-cineole (63.1%), p-cymene (7.7%), α-pinene (7.3%), α-limonene (6.9%), γ-terpinene (3.6%), β-pinene (3.0%)	[21]
Hydrodistillation; GC-MS	α-phellandrene (4.6–10.5%), D-limonene (18.5%), β-myrcene (20.8%), m-cymene (5.0–29.8%), terpinen-4-ol (0.4–4.7%), globulol (3.1–10.5%), spathulenol (4.9–18.8%)	[22]
GC-MS	Bulnesol (19.97%), epi-γ-eudesmol (17.51%), 10-epi-γ-eudesmol (6.93%), valerianol (5.57%), eucalyptol (2.52%)	[23]

**Table 2:** Nanotechnology-enabled systems based on or incorporating *E. globulus*-derived components and their main biologically relevant applications.

Nanotechnology-Based System	Main Physicochemical and Biological Results	Application Relevance	References
Gum Arabic-based nanoencapsulated *E. globulus* oil	GC/GC-MS showed eucalyptol (63.81%) as the major constituent. DLS indicated average particle sizes of about 219 nm for NP and 516 nm for NC systems. The nanoencapsulated oil showed in vitro and in vivo antifungal activity and was effective in controlling anthracnose disease in *Syzygium malaccense* fruits.	Nanoencapsulation for essential-oil stabilization, controlled release, and antifungal delivery	[56]
*E. globulus*-loaded micellar nanoparticles developed in a nanoemulsion system	The optimized formulation used Tween 40 at 9.0 wt.%, producing micelles of 17.13 ± 0.035 nm, with pH 6.57 and viscosity of about 2.3 cP. In vivo, transdermal administration prolonged hot-plate response time, reaching 40.75 s at 60 min, supporting analgesic efficacy.	Transdermal nanocarrier system for analgesic delivery	[57]
O/W nanoemulsions based on eucalyptus essential oil and loaded with azole drugs (fluconazole, itraconazole, ketoconazole)	Nanoemulsion droplet size ranged from 245 to 415 nm, zeta potential from −9.2 to −25.4 mV, and encapsulation efficiency from 40 to 50%. The formulations remained stable for 60 days and showed anti-aspergillosis and anti-mucormycosis activity, while drug release followed the Korsmeyer–Peppas model.	Nanoemulsion-based antifungal drug delivery	[58]
*E. globulus* essential oil-based O/W nanoemulsions	GC–MS showed 1,8-cineole (67.14%), α-pinene (7.50%), and α-terpineol (7.08%) as major constituents. Tween 80 systems were nearly monodispersed (PDI < 0.3) and more stable than Span 80 systems. The nanoemulsions showed ovicidal, larvicidal, and repellent activity against *Spodoptera litura*, with an ovicidal LC50 of 22.331 mg/mL.	Stable nanoemulsion platform for enhancing essential oil bioefficacy	[59]
Chitosan nanoparticles crosslinked with nano-encapsulated *E. globulus* essential oil	The nanoparticles had sizes of about 25–30 nm, zeta potential values between +18 and +27 mV, loading capacity of 15–26%, and encapsulation efficiency of 98–99%. Repellency against ticks was reported as 90%, 80%, 60%, and 50% across successive time intervals.	Biopolymer-based encapsulation for sustained repellent activity	[60]
ZnO nanoparticles biosynthesized from *E. globulus* essential oil	XRD confirmed a pure ZnO phase with an average crystallite size of 24 nm, while DLS indicated a mean size of about 40 nm. The ZnO NPs showed antimicrobial activity against all tested microorganisms, with a maximum inhibition zone of 19.35 ± 0.45 mm for *Klebsiella pneumoniae* at 100 μg/mL. Biofilm inhibition reached 85% against *S. aureus* and 97% against *P. aeruginosa*.	Nano-enabled antimicrobial and antibiofilm system	[61]
Silver nanoparticles (AgNPs) biosynthesized using *E. globulus* aqueous leaf extract	UV–Vis showed an absorption peak at 408 nm, confirming stable AgNP formation. TEM revealed mainly spherical particles with a mean size of 17.5 ± 5.89 nm. The AgNPs displayed stronger antioxidant and antimicrobial activity than the corresponding plant extract.	Plant-extract-mediated antimicrobial and antioxidant nanoplatform	[62]

## Data Availability

No new experimental datasets were generated during the preparation of this review. Because this manuscript was designed as a bibliometric-assisted narrative review rather than a full bibliometric study, the bibliometric dataset was used only for thematic mapping. The underlying search strategy, keyword combinations, and PubMed-derived dataset used for the VOSviewer keyword co-occurrence analysis are available from the corresponding author upon reasonable request.

## Abbreviations

The following abbreviations are used in this manuscript:

Aβ	amyloid-beta
AgNPs	silver nanoparticles
AO/PI	acridine orange/propidium iodide
APP/PS1	amyloid precursor protein/presenilin-1
CLSI	Clinical and Laboratory Standards Institute
DAT	dopamine transporter
DLS	dynamic light scattering
EGEO	*Eucalyptus globulus* essential oil
GC–MS	gas chromatography–mass spectrometry
GPx	glutathione peroxidase
GPX4	glutathione peroxidase 4
GSH	reduced glutathione
hACE2	human angiotensin-converting enzyme 2
HPLC	high-performance liquid chromatography
IC50	half-maximal inhibitory concentration
IL	interleukin
LPS	lipopolysaccharide
MDA	malondialdehyde
MIC	minimum inhibitory concentration
MIC50	minimum inhibitory concentration required to inhibit 50% of the tested microorganisms
MRSA	methicillin-resistant *Staphylococcus aureus*
NC	nanocapsule
NIH/3T3	NIH Swiss mouse embryo fibroblast cell line 3T3
NP	nanoparticle
Nrf2	nuclear factor erythroid 2-related factor 2
O/W	oil-in-water
PDI	polydispersity index
PTGS2/COX-2	prostaglandin-endoperoxide synthase 2/cyclooxygenase-2
ROS	reactive oxygen species
SARS-CoV-2	severe acute respiratory syndrome coronavirus 2
SOD	superoxide dismutase
TEM	transmission electron microscopy
TH	tyrosine hydroxylase
TNF-α	tumor necrosis factor-alpha
UV–Vis	ultraviolet–visible spectroscopy
VRE	vancomycin-resistant enterococci
XRD	X-ray diffraction
ZnO	zinc oxide
